# Supplementary material for: Effect of sex/gender on obesity traits in Canadian first year university students: The GENEiUS study
Source: PLoS One. 2021 Feb 16;16(2):e0247113. doi: 10.1371/journal.pone.0247113 (PMC7886219; doi:10.1371/journal.pone.0247113)
Supplement: S1 Table — (DOCX) [file pone.0247113.s002.docx]

**S1 Table.** Distribution of demographic characteristics in the overall sample (n=245) and in the male (n=48) and female (n=197) subgroups

|  |  | **Overall Sample**  N (%) | **Male Subgroup**  N (%) | **Female Subgroup**  N (%) |
| --- | --- | --- | --- | --- |
| **Race/Ethnicity** | East Asian | 76 (31%) | 12 (25%) | 64 (32.5%) |
|  | White-Caucasian | 61 (24.9%) | 8 (16.6%) | 53 (26.9%) |
|  | South Asian | 46 (18.8%) | 14 (29.2%) | 32 (16.2%) |
|  | Middle-Eastern | 17 (6.9%) | 6 (12.5%) | 11 (5.6%) |
|  | Mixed | 31 (12.7%) | 6 (12.5%) | 25 (12.7%) |
|  | Other | 14 (5.7%) | 2 (4.2%) | 12 (6.1%) |
| **Living Arrangement** | On-campus Residence | 170 (69.4%) | 33 (68.8%) | 137 (69.5%) |
|  | Off-campus student housing | 26 (10.6%) | 5 (10.4%) | 21 (10.7%) |
|  | At home with family | 48 (19.6%) | 9 (18.8%) | 39 (19.8%) |

Data are expressed in counts (N) and percentages (%); Living Arrangement data not collected for one male participant
