# Supplementary material for: Effect of sex/gender on obesity traits in Canadian first year university students: The GENEiUS study
Source: PLoS One. 2021 Feb 16;16(2):e0247113. doi: 10.1371/journal.pone.0247113 (PMC7886219; doi:10.1371/journal.pone.0247113)
Supplement: S2 Table — (DOCX) [file pone.0247113.s003.docx]

**S2 Table.** Sex-specific trends in obesity traits from the beginning to the end of first year by male (n=48) and female (n=197) subgroups

|  | | **Beginning**  Mean (SD) | **End**  Mean (SD) | **Change**  MD (95% CI) | **Time*** |
| --- | --- | --- | --- | --- | --- |
| **Body Weight (kg)** | Males | 71.37 (12.68) | 73.27 (13.12) | 1.90  (1.13 – 2.68) | **<0.001** |
|  | Females | 57.76 (10.18) | 59.22 (10.53) | 1.46  (1.12 – 1.80) | **<0.001** |
| **BMI (kg/m^2^)** | Males | 22.62 (3.79) | 23.36 (3.90) | 0.74  (0.48 – 1.00) | **<0.001** |
|  | Females | 21.25 (3.18) | 21.87 (3.28) | 0.62  (0.49 – 0.76) | **<0.001** |
| **Waist Circumference (cm)** | Males | 81.38 (9.28) | 83.14 (9.83) | 1.76  (0.66 – 2.85) | **0.006** |
|  | Females | 73.55 (7.83) | 74.59 (7.93) | 0.99  (0.41 – 1.58) | **<0.001** |
| **Hip Circumference (cm)** | Males | 100.56 (7.57) | 101.64 (7.19) | 1.08  (0.29 – 1.88) | **0.006** |
|  | Females | 96.36 (7.56) | 97.25 (7.26) | 0.89  (0.46 – 1.32) | **<0.001** |
| **WHR** | Males | 0.808 (0.040) | 0.816 (0.052) | 0.0085  (-0.0027 – 0.0197) | 0.173 |
|  | Females | 0.763 (0.048) | 0.767 (0.049) | 0.0030  (-0.0024 – 0.0084) | 0.193 |

Data are expressed as mean (SD) and mean difference (95% CI); WC data not collected for one participant; Abbreviations: BMI, body mass index; WHR, Waist to hip ratio; MD, Mean difference.*****Non-parametric pairwise comparison stratified by sex (non-adjusted comparison of change in outcomes from beginning to end of school year in male and female subgroups). P-values below 0.05 represented in bold font.
