# Supplementary material for: Effect of sex/gender on obesity traits in Canadian first year university students: The GENEiUS study
Source: PLoS One. 2021 Feb 16;16(2):e0247113. doi: 10.1371/journal.pone.0247113 (PMC7886219; doi:10.1371/journal.pone.0247113)
Supplement: Attachment — Submitted filename: Response to Reviewers.docx [file pone.0247113.s004.docx]

We would like to thank the editor and the reviewers for their exceptional input and suggestions on the article. We have addressed their comments to the best of our ability and we think that the revised version of the manuscript has significantly improved.

Reviewer #1

1- Method: Study is well designed and method is appropriate. However, there is too much explanation about statistical analysis. This section can be written more concise. Instead, previous paper like Beaudry et al, (2019) can be cited.

We thank the reviewer for this comment. We acknowledge the reviewer’s perspective regarding the statistical methods being slightly explanation-heavy for some readers, and hence have made an effort to shorten the section slightly. However, most of the details included in that section have been retained as we feel they are necessary for a few different reasons. Firstly, the submission guidelines of *PLOS One* particularly require detailed reporting of statistical methods as to allow for replication of analysis by anyone who may be interested. As such, many of the details included in this section are based on the reporting criteria specified by the journal. Secondly, in this case, while we followed the methodology of Beaudry et al (2019) given the follow-up nature of our investigation, we further included a couple of additional elements of analysis in our paper (e.g. analyzing differences in obesity traits between males and females at baseline) that were not present in the previous paper by Beaudry and colleagues. Hence, our methods include a couple of additional aspects that are not present in the previous paper and thus require additional explanation. Lastly, while the reviewer is correct in mentioning that we can cite the paper by Beaudry et al. for all the overlapping methodologies between the two papers, we feel that this could affect the readability of our paper. Generally, when methods are simply referenced without inclusion of any details in the paper, it requires an extra effort on the part of the readers to go through a separate referenced and can hence create extra steps for readers. Thus, while we did reference the paper by Beaudry et al. (2019) for readers who may be interested in reviewing that source, we also included some of the fundamental methodological details in our paper in order to provide clarity and sufficient information for the average reader to understand the paper without having to consult additional sources.

2- Results: Results are clear and well organized. However, some data in the text (lines 195-203) are duplicated in table 1. Authors can choose to present them in either text or the table.

We thank the reviewer for the comment. We re-reviewed that section and feel that it is important to include some of the important details in the text even if those details are available in the table. In this case, we chose to only reiterate the information pertaining to the ‘change in the obesity traits.’ Given that the ‘change in traits’ is central focus of our paper and a critical point of discussion later in the paper, we think that mentioning those results in the text, in addition to the table, is important to make sure that readers can take note of that information and keep that important information in mind as they read the paper.

Reviewer #2

1. Demographics: I do not agree with the claim that the study recruited a "multi-ethnic" sample. The categories used to group participants by race and ethnicity might not capture their actual cultural and ethnic backgrounds. Also, your study would benefit from a table that includes the key demographic characteristics in your sample.

We thank the reviewer for the comment. We agree with the reviewer that the composition of our sample may not be reflective of a truly ‘multi-ethnic’ sample that incorporates all the diverse cultural and ethnic backgrounds. We have now removed the term multi-ethnic as a description of our sample. Additionally, we have included a supplementary table with the key demographic characteristics of the sample as per the reviewer’s suggestion.

2. Moderator / grouping variable: Sex was a key study indicator in your study. Yet, females (n=197) outnumbered males (n=48) 4 times over. Your acknowledgement is valid, but it still makes it difficult to make inferences grouping the data in such a way (pg. 17, ln 346-349).

Thank you for the comment. We agree with the reviewer that this is a limitation of the investigation and as mentioned by the reviewer, we have acknowledged this in the discussion section of our paper. As we discussed in the paper, most studies in this area present a similar imbalance in ratio of males and females, including the previous study by Beaudry et al (2019) that we followed. We have now added an additional sentence in the discussion section to further indicate that this imbalance in ratio of males to females is an important limitation that should be considered when interpreting the results of our study.

“Lastly, we acknowledge that our sample had a significant imbalance in the ratio of male to female participants (approximately 20:80). This imbalance in the sex ratio, along with insufficient power for detection of small effects, may have prevented us from detecting subtle sex differences in anthropometric change. However, it is important to note that most previous studies within this context have included a disproportionately larger percentage of female participants [15]. Furthermore, previous Canadian reports have shown varied results and our study results are at least consistent with some of those previous reports. Nevertheless, we recognize this is an important limitation that restricts our ability to make inferences with the results of this investigation.”

3. Small-to-no significant effect sizes: I am wondering if your investigation could in a new direction after learning from failure to reject your null hypothesis. I am optimistic that such a finding is a meaningful finding, which perhaps leads to sub-group analyses or studies with more depth. For example, how about collecting information on the processes that might be shaping weight gain by sex/gender? What are some differences and similarities that might explain each group's weight gain? A qualitative study might be a key next step.

Thank you for the comment. In this case, since we did not find a difference in anthropometric change between males and females, our investigation did not merit further investigation into the different mechanisms of weight gain by sex/gender. Nevertheless, while this is beyond the scope of our paper, we do agree that this is an interesting field of research. We have now added this point as a recommendation for future studies in the discussion section of our paper.

“Future studies may also consider investigating the mechanisms of weight gain in the undergraduate student population by sex/gender, through quantitative and qualitative approaches.”

Reviewer #3 

1. In the paper, the weight gain that occurs during the period studied seems to be a consequence of the university stay (change of environment and habits, sedentary lifestyle ...). However, it is not clear whether this weight change also occurs in the general population of similar age. A global significant weight increase has been described during adulthood. Therefore, this Reviewer thinks that it would be important to compare the data obtained in this study with those that can be found in general population. It would be interesting to have some group of non-university people available to compare, but since this is not the case, it is at least necessary to make the comparison with data available in Ontario from general population.

The reviewer brings up an excellent point. While we were not able to specifically find data pertaining to non-university Canadians in the same age group, we have added a broader comparison to the general Canadian population based on data available from Statistics Canada.

“Nevertheless, this represents a significant change when compared to the general Canadian population. A report from Statistics Canada, involving data collected through the Canadian National Health Survey, previously indicated an average weight gain of 0.5 to 1 kg over a two-year period among Canadian adults [36]. Hence, in comparison, an average weight gain of 1.55 kg over a 5-month period among first-year university students represents a noteworthy change.”

2. Since it is possible that part of the differences found between studies could be caused by the ethnic distribution of the students, I do not know if it would be possible to subdivide, at least the group of women, more numerous, thus comparing if there are differences associated with ethnic distribution.

Thank you for the great comment. We acknowledge that examining the differences associated with ethnic distribution would be an interesting area of analysis. However, given our limited sample size in this case, we feel that an analysis of ethnic subgroups within the sex/gender subgroups would be severely underpowered. We think this analysis would also not be adequately powered with male group as our sample includes participants from multiple ethnic groups with the distribution of participants across each of the ethnic subgroups being unequal. Hence, the number of people within each ethnicity x sex subgroup is minimal and hence such analysis would be severely underpowered. Nonetheless, we agree with the reviewer that this is an interested area of investigation, and while it may not be possible to include the suggested analysis in this report due to power and sample size limitations, we have another paper that is currently in revision at PLOS One that independently examines the effect of race/ethnicity on anthropometric traits in the same cohort.

Reviewer #4

1. Throughout the manuscript, the authors dive into the idea of obesity and the potential for the 'freshman 15' to contribute to obesity development. While this is likely true, the authors only briefly in the discussion touch on the possibility for continued development. This would be particularly true for males as many can undergo late-stage development during their early college years. Therefore, its unclear if the increase in weight gain for males is truly a reflection of an obesogenic environment or development.

We thank the reviewer for this important comment. We have now added a paragraph in the discussion section of the paper that addresses these points.

“It is important to note here that the weight gain observed in our sample may not be entirely attributed to an increase in fat, but also to additional contributing factors such as continued development and increase in muscle mass. Unfortunately, in this case, we could not evaluate parameters such as lean mass or fat mass. Nonetheless, our investigation of adiposity indicators, such as waist and hip circumference, revealed significant increases in those areas among both male and female participants. Hence, based on our data, we postulate that one of the components contributing to the observed weight gain in our sample may possibly be a potential increase in fat. However, we acknowledge that there may be additional contributing factors as discussed above, and recognize that the data is limited in terms of the information it provides to characterize the observed change.”

2. In addition, without the knowledge of physical activity among these individuals, we again can not be sure if the weight gain is purely fat mass or a combination of fat mass and muscle mass (particularly if PA was increased). Again this is not likely, but should be more thoughtfully developed in the discussion.

The reviewer brings up a fair point. We have now extended the discussion about the limitations pertaining to the characterization of weight change in our study, including the lack of information about physical activity.

“Additionally, we did not investigate physical activity as a covariate in our models, as done by Beaudry *et al.* in their study, due to a change in our method of measurement after the two first waves of recruitment. Furthermore, we did not examine body composition parameters in our study and hence could not specifically characterize the observed anthropometric change. It is important to note here that the weight gain observed in our sample may not be entirely attributed to an increase in fat, but also to additional contributing factors such as continued development and / or increased physical activity, and increase in muscle mass. Unfortunately, in this case, we could not evaluate parameters such as lean mass or fat mass. Nonetheless, our investigation of adiposity indicators, such as waist and hip circumference, revealed significant increases in those areas among both male and female participants. Hence, based on our data, we postulate that one of the components contributing to the observed weight gain in our sample may possibly be potential increase in fat. However, we acknowledge that there may be additional contributing factors as discussed above, and recognize that the data is limited in terms of the information it provides to characterize the observed change.”

3. Lastly, this group largely falls within the realm of normal weight status with only a small percentage falling in the category of overweight and an even smaller percent falling in the category of obese. This is important to note as it may not reflect other Universities, for instance those in the south in the USA where obesity rates can be over 40%. Again, are these students obesogenic?

We agree with the reviewer that there are differences in university environments. We talked about campus heterogeneity as an important factor through the discussion section of our paper. We have further added a note about the differing distribution of BMI weight status across universities.

“Additionally, the baseline distribution of BMI weight status at a university can also impact the trajectory of BMI change. Hence, within this context, our differing results highlight the importance of conducting multiple studies not only across Canada but also within each province because multiple factors may differentiate university campuses from each other.”

Apart from that, we have also added a sentence in the results section to highlight the distribution of BMI weight statuses observed in our sample by the end of the academic year.

“In terms of their BMI categories, by the end of the academic year 77.1% (n = 189) of the participants were in the normal weight range, 8.6% were underweight (n = 21), 11.4% were overweight (n = 28), and 2.9% (n = 7) were obese.”

Reviewer #5

1.- Statistical methods: it is not clear why authors choose to use the non-parametric tests for baseline and pairwise comparison of outcomes (Tables 1 and 2); while using the RMANOVA test for other year- and sex-specific outcomes. I suggest that outcomes are analyzed for their parametric or non-parametric distribution, justifying and homogenizing the choice of the statistical tests used.

Thank you for the comment. In this case, given that our investigation was a follow-up to a previous report by Beaudry et al (2019), we tried to largely follow the same analytical methodology as the paper by Beaudry et al. (i.e. RMANVOA). Nevertheless, we further included a couple of additional elements of analysis in our paper (e.g. analyzing differences in obesity traits between males and females at baseline) that were not present in the previous paper by Beaudry and colleagues. Particularly, we used non-parametric tests for evaluation of traits without adjustment for covariates and used the RMANOVA test with transformation for analysis of traits with adjustment for covariates. We recognize that there are different analytical approaches that be used to analyze the data. In fact, we used a different approach involving regression analysis for our other papers on the effect of living arrangement and ethnicity on anthropometric that are currently published (Sharma et al., PLOS One 2020) or under revision. In this case, we feel that the respective tests used here are appropriate, and do not see the need to homogenize the approach.

2.- Authors claim several times throughout the text as a relevant finding that males presented with larger BW, WC or HC than females, while these parameters are usually larger in males than in females. In my opinion, only the higher BMI and WHR in males is a relevant finding, showing a stronger tendency to obesity in males.

Thank you for the comment. While we agree with the reviewer that BMI and WHR are critical parameters and could be the focus of a paper, we feel that it is still important to discuss the other parameters in the text as well considering that the data for these traits have been presented in the paper. Given that we conducted this investigation as a follow-up to the previous paper by Beaudry et al, we assessed and discussed the same traits as the ones discussed by Beaudry and colleagues. Additionally, in recent times, there has generally been an increasing amount of the literature on the prognostic value of indicators such as WC and hence including these results may be of interest to the readers.

3.- Since the mean topic of the work is the difference of sex in weight gain during first-year university, the ratio male/female in the cohort is a very important piece of information. However, it is not shown until the Results section. Here, authors report that 80.4% of the sample were female, but it is not clear to what cohort is this % referring to. Since it is presented in the same paragraph where the initial recruited population is described, (n=361), I understand that it 80.4% of this population are females. Therefore, only 76 males were included in the study. However, in Table 2 authors indicate a 19.6% (n=48) males, so maybe the 80.4% females indicated earlier in the Results section referred to the population that completed the follow-up. This piece of information (% of females/males at every stage of the study) is very important for this precise work but is not clearly shown in the text until the final part of the Discussion section. I think that it should be emphasized and made clear from the beginning, including the abstract. Also, and related to this, the ethnicity and living arrangements were only shown for the total population, but not indicated for each sex, which could also shed some light to the differences.

This is an excellent point. We have now updated the text to better reflect the cohort information and have included a supplementary table the described the ethnicity and living arrangement distribution by sex/gender.

4.- Tables 1 and 2: indicate the precise statistical methods used for these tables. I assume, from the Methods section, that the Wilcoxon signed-rank test was performed for Table 1, and the Mann-Whitney U test was used for Table 2, since Table 1 shows the paired data and Table 2 shows the baseline. But I think it should be stated in the legends for clarity.

The reviewer brings up an important point. We have now updated the legends of both the tables as per the reviewer’s recommendation.

5.- In Table 3 there are a few unclear notes (1, 2), that are hard to find in the table text. In particular, it is not clear to me why only BW, WC and WHR were calculated using living arrangement as a covariate, and BMI also used cohort as covariate. Also, it is not clear to me the nature of the “cohort” covariate, a clear explanation would be welcome. Also it would be interesting to see if ethnicity was a relevant covariate. I assume that, since it was not included, it was not; but I would mention it in the text. Author indicate ethnicity as a possible reason for the discrepancy between this study and previous ones; but the data presented in this study does not indicate that ethnicity is a relevant covariate.

Thank you for the comment. In this case, we used the same statistical methodology and covariate adjustment strategy as the previous paper by Beaudry et al (2019). As such, in accordance with the protocol of Beadry et al, the covariates were only retained in the model if their interaction with the main effect (i.e. time) was significant or marginally significant. We have included this detail in the statistical methods section and have included a reference to the paper by Beaudry et al. (2019) for readers who are interested in reading their protocol in further detail. Cohort refers to the cohort of recruitment in this case. We have updated the legend of Table 3 to better reflect this. While we agree that ethnicity is an interesting variable to test, we did not explore it as a covariate in this case particularly because it was not also included as a covariate by Beaudry et al. in their paper. Hence, in order to keep the methodology consistent, we only explored the variables that were evaluated in the previous paper. However, we so have another paper that is currently in revision at PLOS One that examines the effect of ethnicity on anthropometric traits in GENEiUS.

6.- Authors mention in the Discussion section an increase in BMI with no increase in WHR; a deeper discussion of the nutritional and metabolic meaning of this finding would be welcome.

We thank the reviewer for this inspired comment. We have now added a paragraph in the discussion section of the paper that addresses these points.

“It is important to note here that the weight gain observed in our sample may not be entirely attributed to an increase in fat, but also to additional contributing factors such as continued development and increase in muscle mass. Unfortunately, in this case, we could not evaluate parameters such as lean mass or fat mass. Nonetheless, our investigation of adiposity indicators, such as waist and hip circumference, revealed significant increases in those areas among both male and female participants. Hence, based on our data, we postulate that one of the components contributing to the observed weight gain in our sample may possibly be a potential increase in fat. However, we acknowledge that there may be additional contributing factors as discussed above, and recognize that the data is limited in terms of the information it provides to characterize the observed change.”

7.- Full data are not available in a public repository, and there is no Data availability statement in the text. Please, provide a link to download the full, anonymized data in a public repository.

Thank you for bringing up this important point. The dataset has now been included.
