# Supplementary material for: Effect of sex/gender on obesity traits in Canadian first year university students: The GENEiUS study
Source: PLoS One. 2021 Feb 16;16(2):e0247113. doi: 10.1371/journal.pone.0247113 (PMC7886219; doi:10.1371/journal.pone.0247113)
Supplement: Attachment — Submitted filename: Response to reviewers.docx [file pone.0247113.s006.docx]

We would like to thank the editor and reviewer 5 for their feedback on the second revision of our article. We have addressed their comments below to the best of our ability, and we think that the manuscript is now mature for publication.

Reviewer #5: In this work, Sharma and colleagues perform a longitudinal study investigating the morphometric evolution of first year University students in the McMaster University (Ontario, Canada). During this first year, several previous reports had shown a significant increase in BW and BMI. Some of them had reported significant sex-specific trends in these parameters, although the existing literature was heterogeneous and not conclusive. This new study, although including a low number of individuals (245), confirms a significant increase in BW, BMI, WC and HC in the students, with no significant difference between sexes, although males showed a tendency to greater increases in BW, BMI, WC and HC. This work, although limited, adds to previous reports using a longitudinal and relatively more complete assessment of morphometric parameters, and could be of use for future meta-analysis.

We would like to thank the reviewer for the constructive comment.

I have several comments:

1.- Statistical methods: it is not clear why authors choose to use the non-parametric tests for baseline and pairwise comparison of outcomes (Tables 1 and 2); while using the RMANOVA test for other year- and sex-specific outcomes. I suggest that outcomes are analyzed for their parametric or non-parametric distribution, justifying and homogenizing the choice of the statistical tests used.
A: Thank you for the comment. In this case, given that our investigation was a follow-up to a previous report by Beaudry et al (2019), we tried to largely follow the same analytical methodology as the paper by Beaudry et al. (i.e. RMANVOA). Nevertheless, we further included a couple of additional elements of analysis in our paper (e.g. analyzing differences in obesity traits between males and females at baseline) that were not present in the previous paper by Beaudry and colleagues. Particularly, we used non- parametric tests for evaluation of traits without adjustment for covariates and used the RMANOVA test with transformation for analysis of traits with adjustment for covariates. We recognize that there are different analytical approaches that be used to analyze the data. In fact, we used a different approach involving regression analysis for our other papers on the effect of living arrangement and ethnicity on anthropometric that are currently published (Sharma et al., PLOS One 2020) or under revision. In this case, we feel that the respective tests used here are appropriate, and do not see the need to homogenize the approach.
R: it sounds odd that the only argument to justify the use of a statistical method is “that others also used it before”, rather than proving that the statistical method is the most appropriate for the kind of data analyzed.
A2: Thank you for the comment. We agree “that others also used it before” would not be a sufficient argument to apply the same approach. However, it seems the reviewer overlooked the other arguments we provided to justify our approach. Firstly, we think that the statistical methods proposed by Beaudry et al. are adequate to answer the questions asked. Secondly, the manuscript by Beaudry et al. has been reviewed and validated by experts, as part of the peer-review process, so we are not the only ones to think that the statistical approach used by Beaudry et al. is adequate. Thirdly, given the follow-up nature of our investigation, using a drastically different statistical approach may have added heterogeneity and impaired our ability to compare our results to those of Beaudry et al. Fourthly, we have provided additional elements of analysis in our paper that were not present in Beaudry’s paper and hence those methods differ –particularly with respect to using non-parametric tests for a raw comparison of traits in the absence of covariates, and the using RMANOVA, as done by Beaudry et al. for the comparison of traits with adjustment for covariates. We believe these are adequate reasons to justify our approach. However, we understand well the reviewer’s concern and we have added a limitation in the discussion to reflect their important point. “In this investigation, we followed the analysis protocol outlined by Beaudry at al. to optimize our ability to compare our results. However, we are aware that alternative statistical methods can also be utilized to analyze this data.
R2: I leave this matter for the Editor to decide. I still think that an evaluation of the parametric and non-parametric type of data would support the correct use of the chosen statistical methods, and would complete the information presented in the paper. Although I also acknowledge the reasons given by the authors for not changing the statistical methods from Beaudry et al., still if the data are not suited for a precise statistical method, using it is incorrect and could help fixing a previous mistake.

Thank you for the feedback. Once again, we respectfully disagree with the reviewer that performing non-parametric analyses on untransformed longitudinal data adjusted for covariates is needed in our study, and that for different reasons:

1) The statistical methods proposed by Beaudry et al. are adequate to answer the questions asked. Anthropometric data are skewed and depart systematically from normality. Performing RMANOVA on non-normal longitudinal data following its inverse normal rank transformation is the best analytical strategy to deal with the lack of normality of data.
2) The previous publication by Beaudry et al. has been published in *PLOS One* and has been validated by an academic editor and at least two reviewers. This is a strong indication that the statistical approach used by Beaudry et al. and more recently by us is adequate. If the reviewer really thinks that the statistical design in the Beaudry et al. publication is inadequate, their first action may be to post a comment on the *PLOS One* website and ask for a formal correction of the article. We checked the Beaudry et al. publication on the website and since its publication 1.5 years ago, no comment has been posted regarding the Baudry’s publication. Knowing that the article has been viewed by 5,800 people, the absence of negative comment strongly indicates that the reader’s community is comfortable with the statistical methods used in the paper.
3) Given the follow-up nature of our investigation, using a drastically different statistical approach may have added heterogeneity and impaired our ability to compare our results to those of Beaudry et al.
4) We have provided additional elements of analysis in our paper that were not present in Beaudry’s paper and hence those methods differ –particularly with respect to using non-parametric tests for a raw comparison of traits in the absence of covariates. As we use both parametric an non-parametric statistical analyses in our study, we do not understand why the reviewer is not satisfied.
5) The method used by Beaudry et al. (RMANOVA with transformed data) is by far the most frequently used method in literature when it comes to analyzing longitudinal series of non-normal data with adjustment for covariates. As the reviewer did not provide any guidance on the type of statistical tests he wanted us to use, we had to make an extensive literature search to find examples of non-parametric tests that allow the analysis of longitudinal series of untransformed non-normal data with adjustment for covariates. Unfortunately, we did not find any alternative non-parametric method that we apply to our study. This indicates that the reviewer’s request is very unconventional.

We feel that the reviewer’s repeated request is kind of odd and is very challenging to address in practice. This is the third time we provide a strong justification for our analytical design, and we do not understand why the reviewer is not more receptive to our arguments. We leave this matter for the Editor to decide, but we hope he is satisfied with our answers. We have added a limitation in the discussion to reflect the reviewer’s comment. “In this investigation, we followed the analysis protocol outlined by Beaudry at al. to optimize our ability to compare our results. However, we are aware that alternative statistical methods may also be utilized to analyze this data.


2.- Authors claim several times throughout the text as a relevant finding that males presented with larger BW, WC or HC than females, while these parameters are usually larger in males than in females. In my opinion, only the higher BMI and WHR in males is a relevant finding, showing a stronger tendency to obesity in males.
A: Thank you for the comment. While we agree with the reviewer that BMI and WHR are critical parameters and could be the focus of a paper, we feel that it is still important to discuss the other parameters in the text as well considering that the data for these traits have been presented in the paper. Given that we conducted this investigation as a follow-up to the previous paper by Beaudry et al, we assessed and discussed the same traits as the ones discussed by Beaudry and colleagues. Additionally, in recent times, there has generally been an increasing amount of the literature on the prognostic value of indicators such as WC and hence including these results may be of interest to the readers.
R: I think my point was not well understood. I agree that analyzing changes in BW, WC and HC of individuals with time is a very valuable analysis. What I do not agree is on the treatment as a relevant finding of the difference between male and female volunteers in BW, WC and HC: males always display larger BW, WC and HC in average than females, that is not biologically relevant.
A2: Thank you for the clarification. While we agree with the reviewer that the observation that males always display larger BW, WC and HC on average is a well-established observation in literature, we still feel it is important to include this finding in our report for a couple of reasons. Firstly, we do think that males being larger than females is a biologically important observation. Secondly, in any case, we believe that before discussing the ‘change’ in traits across groups, it is imperative that the baseline distribution of the traits are assessed and reported, as knowing the baseline distribution provides context to better interpret values of change. In this case, while this may seem redundant given the extensive amount of literature on sex-based anthropometric patterns, we believe it is still important that we confirm this finding in our sample and provide that baseline context for the readers before providing a discussion on the magnitude of change observed in these traits. Apart from that, it should be noted that we only discussed this observation very briefly in our result and discussion sections with only a few lines dedicated this observation in the entire manuscript. We are aware that this is a common observation and hence it is definitely not something we have discussed frequently or emphasized as a core point of discussion in our manuscript. In fact, in our brief discussion of this observation, we also particularly acknowledge that this is a well-established observation in different age groups in literature (lines 312-315). Our discussion on the change in traits definitely makes up the majority of our paper.
R2: I agree with the author’s response, thank you.

We would like to thank the reviewer for the constructive comment.

3.- Since the mean topic of the work is the difference of sex in weight gain during first-year university, the ratio male/female in the cohort is a very important piece of information. However, it is not shown until the Results section. Here, authors report that 80.4% of the sample were female, but it is not clear to what cohort is this % referring to. Since it is presented in the same paragraph where the initial recruited population is described, (n=361), I understand that it 80.4% of this population are females. Therefore, only 76 males were included in the study. However, in Table 2 authors indicate a 19.6% (n=48) males, so maybe the 80.4% females indicated earlier in the Results section referred to the population that completed the follow-up. This piece of information (% of females/males at every stage of the study) is very important for this precise work but is not clearly shown in the text until the final part of the Discussion section. I think that it should be emphasized and made clear from the beginning, including the abstract.
Also, and related to this, the ethnicity and living arrangements were only shown for the total population, but not indicated for each sex, which could also shed some light to the differences.
A: This is an excellent point. We have now updated the text to better reflect the cohort information and have included a supplementary table the described the ethnicity and living arrangement distribution by sex/gender.
R: thank you for your efforts. I still do not see any mention to the males/females ratio in the abstract, where I think that it is a very relevant piece of information. Please, indicate where exactly in the text these changes were included, to be able to verify them easily.
A2: The size of the analyzed sample and the gender ratio has now been specified in the abstract (line 29), methods section (lines 120-122), and the results section (line 172,175). Thank you.
R2: Thank you for this clarification.

We would like to thank the reviewer for the constructive comment.

4.- Tables 1 and 2: indicate the precise statistical methods used for these tables. I assume, from the Methods section, that the Wilcoxon signed-rank test was performed for Table 1, and the Mann-Whitney U test was used for Table 2, since Table 1 shows the paired data and Table 2 shows the baseline. But I think it should be stated in the legends for clarity.
A: The reviewer brings up an important point. We have now updated the legends of both the tables as per the reviewer’s recommendation.
R: thank you for your efforts, it is more complete now.

5.- In Table 3 there are a few unclear notes (1, 2), that are hard to find in the table text. In particular, it is not clear to me why only BW, WC and WHR were calculated using living arrangement as a covariate, and BMI also used cohort as covariate. Also, it is not clear to me the nature of the “cohort” covariate, a clear explanation would be welcome.
Also it would be interesting to see if ethnicity was a relevant covariate. I assume that, since it was not included, it was not; but I would mention it in the text. Author indicate ethnicity as a possible reason for the discrepancy between this study and previous ones; but the data presented in this study does not indicate that ethnicity is a relevant covariate.
A: Thank you for the comment. In this case, we used the same statistical methodology and covariate adjustment strategy as the previous paper by Beaudry et al (2019). As such, in accordance with the protocol of Beadry et al, the covariates were only retained in the model if their interaction with the main effect (i.e. time) was significant or marginally significant. We have included this detail in the statistical methods section and have included a reference to the paper by Beaudry et al. (2019) for readers who are interested in reading their protocol in further detail. Cohort refers to the cohort of recruitment in this case. We have updated the legend of Table 3 to better reflect this. While we agree that ethnicity is an interesting variable to test, we did not explore it as a covariate in this case particularly because it was not also included as a covariate by Beaudry et al. in their paper. Hence, in order to keep the methodology consistent, we only explored the variables that were evaluated in the previous paper. However, we so have another paper that is currently in revision at PLOS One that examines the effect of ethnicity on anthropometric traits in GENEiUS.
R: Thank you for your efforts. I understand that only the covariates that interacted significantly (or marginally singifficantly) with the main effect (time) were included. It would be useful to know the exact degree of significance for the analyzed variables, since the term “marginally” significant is not very clear. Also, authors indicate that they did not include ethnicity “because it was not included in Beaudry et al”; again, this is not a valid answer, because this is a different study. Was ethnicity significantly interacting with time? If it was, it should be included; if notit was not, that is a valid argument, not Beaudry’s.
A2: Thank you for the comment. We have now specified our definition of marginal p-value (i.e. p<0.1) in the methods section of the paper for further clarity. With regards to the analysis of ethnicity as a covariate, as discussed previously, we did not initially intend on exploring ethnicity as a covariate as we followed the analytical protocol, covariate adjustment strategy, outlined by Beaudry et al. (2019) in order to minimize the heterogeneity between the study methodologies for better comparability of results. While this is a “different study, we believe that this is still an important consideration as changing the analytical strategy can introduce methodological heterogeneity and consequently reduce comparability, which is not favorable for a follow up investigation like ours. Nevertheless, we acknowledge the reviewer’s point and recognize the merit in assessing ethnicity as a potential covariate. However, in this case, we are unable to assess ethnicity as a covariate in the current investigation as the sample sizes of some of the represented ethnic groups in our study are too small and hence insufficient for adequate statistical adjustment. While our study sample includes certain large homogenous ethnic groups (i.e. East Asian, white-Caucasian, and South Asian), as we have indicated in Supplementary Table 1, the other ethnic groups in our sample are not sufficiently represented in terms of sample size. For example, the Middle Eastern ethnic group contains only 17 participants (6 males, 11 females). Similarly, in our sample there are 14 participants that collectively belong to other ethnic groups (e.g. African, Latin American etc.) with insufficient representation of each ethnic group in our study sample. As such, given that the number of participants in each of the individual ethnic categories are limited, we believe that the conclusions drawn based on such low subgroup sizes would be skewed due to inadequate power. We have now added this as a limitation in the discussion section of the paper to highlight this comment. “We could not investigate ethnicity as a potential covariate in our present analysis due to the limited sample size of certain ethnic subgroups in our overall study sample.”
R2: Thank you for this new information, I think it is clearer now.

We would like to thank the reviewer for the constructive comment.

6.- Authors mention in the Discussion section an increase in BMI with no increase in WHR; a deeper discussion of the nutritional and metabolic meaning of this finding would be welcome.
A: We thank the reviewer for this inspired comment. We have now added a paragraph in the discussion section of the paper that addresses these points.
“It is important to note here that the weight gain observed in our sample may not be entirely attributed to an increase in fat, but also to additional contributing factors such as continued development and increase in muscle mass. Unfortunately, in this case, we could not evaluate parameters such as lean mass or fat mass. Nonetheless, our investigation of adiposity indicators, such as waist and hip circumference, revealed significant increases in those areas among both male and female participants. Hence, based on our data, we postulate that one of the components contributing to the observed weight gain in our sample may possibly be a potential increase in fat. However, we acknowledge that there may be additional contributing factors as discussed above, and recognize that the data is limited in terms of the information it provides to characterize the observed change.”
R: Thank you for the new discussion.

7.- Full data are not available in a public repository, and there is no Data availability statement in the text. Please, provide a link to download the full, anonymized data in a public repository.
A: Thank you for bringing up this important point. The dataset has now been included.
R: Thank you for this dataset, it is very useful.
