# Supplementary material for: Effect of sex/gender on obesity traits in Canadian first year university students: The GENEiUS study
Source: PLoS One. 2021 Feb 16;16(2):e0247113. doi: 10.1371/journal.pone.0247113 (PMC7886219; doi:10.1371/journal.pone.0247113)
Supplement: Attachment — Submitted filename: Response to reviewers.docx [file pone.0247113.s007.docx]

Reviewer #5: There is only one question remaining, all the other comments were properly answered by authors. Congratulations to the authors for their work.

We would like to thank the editor and the reviewer for their feedback on the third revision of our article. We have addressed their comments below to the best of our ability.

This is my original question:
1.- Statistical methods: it is not clear why authors choose to use the non-parametric tests for baseline and pairwise comparison of outcomes (Tables 1 and 2); while using the RMANOVA test for other year- and sex-specific outcomes. I suggest that outcomes are analyzed for their parametric or non-parametric distribution, justifying and homogenizing the choice of the statistical tests used.

I am afraid that there has been a lack of communication with this issue in subsequent answers. The data obtained in the repeated measures of the study are not normal, and this is why authors use non-parametric tests (Mann-Whitney or Wilcoxon signed-rank tests) to analyze these data. However, when analyzing differences in anthropometric outcomes over the year and the effect of sex on anthropometric change, authors perform an inverse normal rank transformation that converts their non-normal data into normal data. This transformed data is proposed to be now suitable for RMANOVA analysis.
Probably this strategy has been used in other reports previously, and I assume that, since transformed data are now normal, the test is correct. I was not aware of the use of this statistical strategy, and this is why I asked. I strongly suggest that authors cite previous similar reports to support this statistical strategy (they mention the existence of several reports, but refer to none in particular).

We thank the reviewer for the clarification. We now mention in the Methods section that the inverse normal rank transformation resulted in the normality of the transformed data distribution. This means that our RMANOVA analysis of these transformed data is valid, as acknowledged by the reviewer. We also cite previous reports where the same transformation has been used.

I understand that using non-parametric tests for the analysis of non-normal repeated measures would have been the best option. There are several of these tests available:
<https://onlinelibrary.wiley.com/doi/abs/10.1002/sim.4780101210>
<https://www.jstatsoft.org/article/view/v050i12>
<https://www.jstatsoft.org/article/view/v064i09>

Actually, normal transformation is a question of debate in the statistical field (<https://www.ncbi.nlm.nih.gov/pmc/articles/PMC2921808/>).

However, I also understand that, if this transformation is a common practice, and it is the strategy followed by the paper that is being used as a model by the authors (Beaudry et al.,), it makes sense to keep the transformation + RMANOVA for this precise analysis, since, strictly speaking, transformed data are normal.

The reviewer makes a relevant point, thank you. We have now added as a limitation in the Discussion that normal transformation is a question of debate in the statistical field.

I have not more to say in this point. I would have acknowledged this simple clarification (transformed data are normal, once transformed) when I first asked. I think we all have learnt with this revision!

Once again, we thank the reviewer for the very helpful clarification.
